# Effects of the Herbicide Atrazine on Crustacean Reproduction. Mini-Review

**DOI:** 10.3389/fphys.2022.926492

**Published:** 2022-06-16

**Authors:** Gabriela R. Silveyra, Daniel A. Medesani, Enrique M. Rodríguez

**Affiliations:** Departamento de Biodiversidad y Biología Experimental, Laboratorio de Fisiología de Crustáceos, Facultad de Ciencias Exactas y Naturales, Universidad de Buenos Aires, CONICET, Instituto de Biodiversidad y Biología Experimental y Aplicada (IBBEA), Ciudad Universitaria, Buenos Aires, Argentina

**Keywords:** agrochemicals, reproductive aspects, invertebrates, ovary, vitellogenin, morphological abnormalities

## Abstract

Atrazine, one of the most intensively applied herbicides worldwide, is commonly found in several water bodies, affecting the associated fauna. Autochthon crustacean species have been relatively less studied, compared to vertebrate species, particularly concerning reproductive success. In this mini-review, we summarize the relevant information about the effects of atrazine exposure on the main reproductive aspects of crustaceans. One of these effects is related to the inhibition of ovarian growth. In this respect, a diminished vitellogenin content was found in the ovary of crabs exposed to atrazine during the entire period of ovarian growth, in correlation with a reduced oocyte size and a delay of ovarian maturation. Similar results were observed in crayfish. Atrazine was also able to affect the reproductive process, acting as an endocrine disruptor. In this sense, this herbicide was suspected to affect the secretion of some neurohormones involved in the gonadal growth, as well as to alter the circulating levels of steroid hormones which promote the synthesis of vitellogenin for ovarian growth. Moreover, atrazine induced sexual differentiation in juvenile crayfish toward a higher proportion of females, while it produced an increment of males in daphnids. Another aspect affected by this herbicide was the reduction of offspring production, as well as several embryonic abnormalities; genotoxic effects have been also reported in crayfish. Finally, some metabolic imbalances, such as reduction in energy reserves, have been observed in some species, together with oxidative stress and histopathological effects.

## Introduction

The herbicide atrazine is intensively applied to control weeds in corn, sorghum, and sugar cane crops. Although some restrictions have been imposed on the use of atrazine in several countries, it remains the second-most used herbicide in the United States after glyphosate. This herbicide has been detected in areas adjacent to fields, reaching watercourses by runoff and thus affecting the associated fauna. Atrazine has been detected in water bodies at concentrations ranging from 0.1 μg/L to more than 100 μg/L after being applied (USEPA 2002). Moreover, in water bodies close to agricultural areas of the United States, atrazine concentrations can be found at levels as high as 1 mg/L, while in Australian soil this herbicide was found at 100 μg/g (Graymore et al., 2001). Although atrazine has not high affinity for absorption on sediments, the fraction associated with this substrate can be significantly high, therefore affecting the ecosystem (Jablonowski et al., 2011; Singh et al., 2017).

There is a bulk of evidence about the harmful effects of atrazine on vertebrate species, especially fish and amphibians. These effects include alteration of steroid hormone levels, e.g., leading to feminization in amphibian males (Hayes et al., 2006), as well as inhibition of the cortisol response in fish (Koakoski et al., 2014). Moreover, teratogenicity, genotoxicity, and oxidative stress have been reported in fish exposed to atrazine (Adeyemi et al., 2015), together with several histopathological effects (Paulino et al., 2012). Comparatively to vertebrate aquatic species, fewer studies have been conducted on crustacean species, concerning the effects of atrazine on reproduction (Rodríguez et al., 2007). Nevertheless, there is enough evidence that shows significant effects of this herbicide on a series of processes related to reproduction, as summarized below.

## Inhibition of Ovarian Growth

One of the most relevant effects of atrazine on crustacean reproduction is the inhibition of ovarian growth. When the crab *Neohelice granulata* was exposed to atrazine during the 3-month pre-reproductive period, during which the ovary is growing out before spawning, a significant (*p* < 0.05) delay in ovarian growth was detected, in terms of a lower proportion of vitellogenic oocytes (Silveyra et al., 2017, Figure 1A). The same effect was observed after 1 month of exposure to atrazine during the ovarian re-maturation, i.e., just after the first spawning, once the reproductive period is attained (Álvarez et al., 2015). Correspondingly, during the 3-month exposure mentioned above, a diminished vitellogenin content was found in the ovary of *N. granulata*, together with a reduced size in both previtellogenic and vitellogenic oocytes (Silveyra et al., 2017).

**FIGURE 1:**
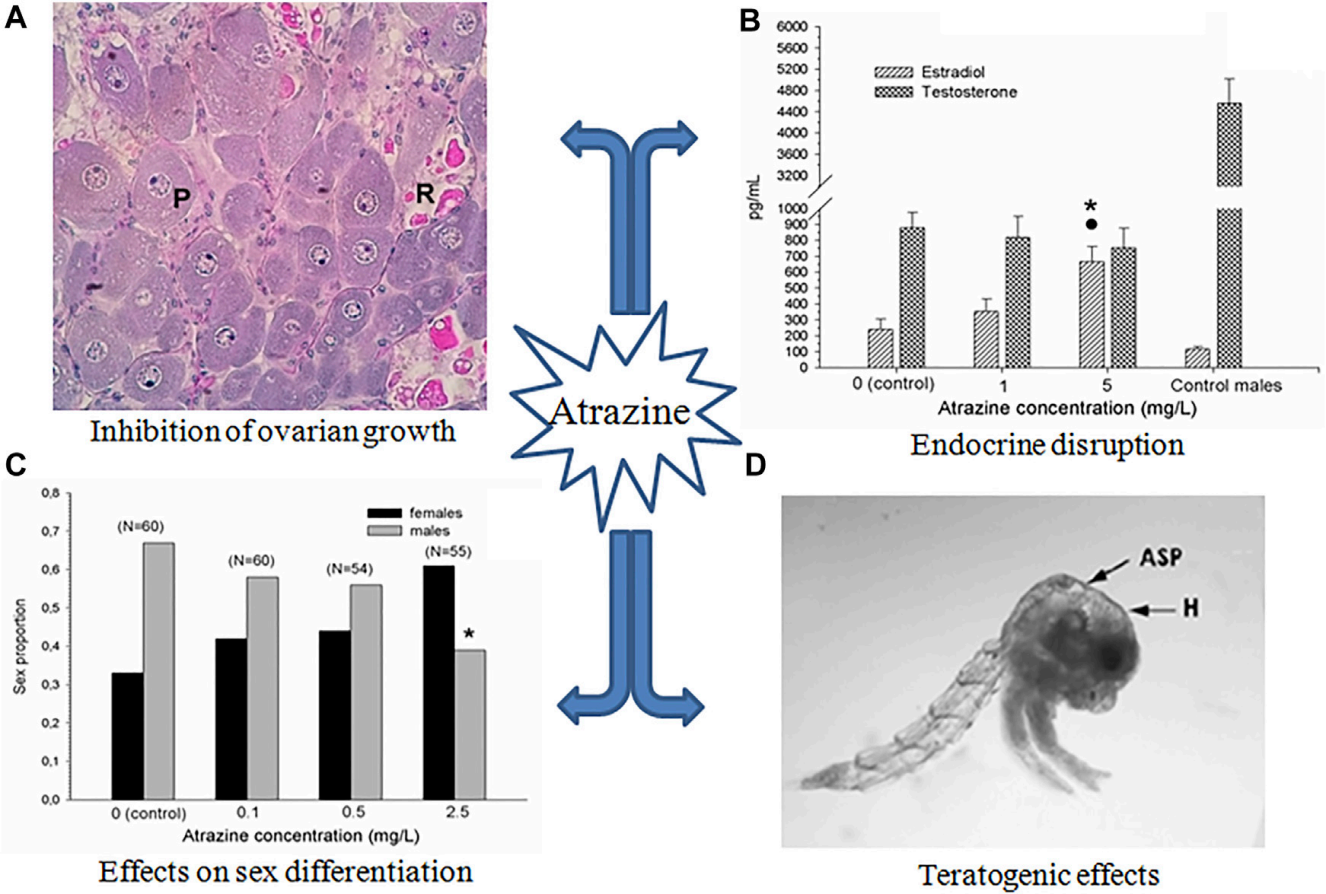
Main effects of atrazine on crustacean reproduction. **(A)** Relative proportion of oocyte types in the ovary of *N. granulata* after 3 months of exposure (P: previtellogenic, and R: reabsorbed vitellogenic oocytes; no normal vitellogenic oocytes developed). Modified from Silveyra et al. (2017). **(B)** Changes in steroid in the hemolymph of *Procambarus clarkii* after 1 month of exposure. Asterisk and dot indicate significant differences (*p* < 0.05) with respect to control and the lowest concentration, respectively; control male data are also included for comparative purposes. From Silveyra et al. (2019). **(C)** Proportion of sex in early juveniles of *Cherax quadricarinatus* exposed during 4 weeks. Asterisk indicates significant differences (*p* < 0.05) with respect to control; number of animals is indicated between brackets. From Mac Loughlin et al. (2016). **(D)** zoea I from hatched from ovigerous females of *N. granulata* exposed during the entire egg incubation period (H: hydropsy; ASP: atrophied dorsal spine; melanization, atrophy of setae, and atrophied eyes can be also observed). Modified from Álvarez et al. (2015). All figures are reproduced with permission.

**FIGURE 2:**
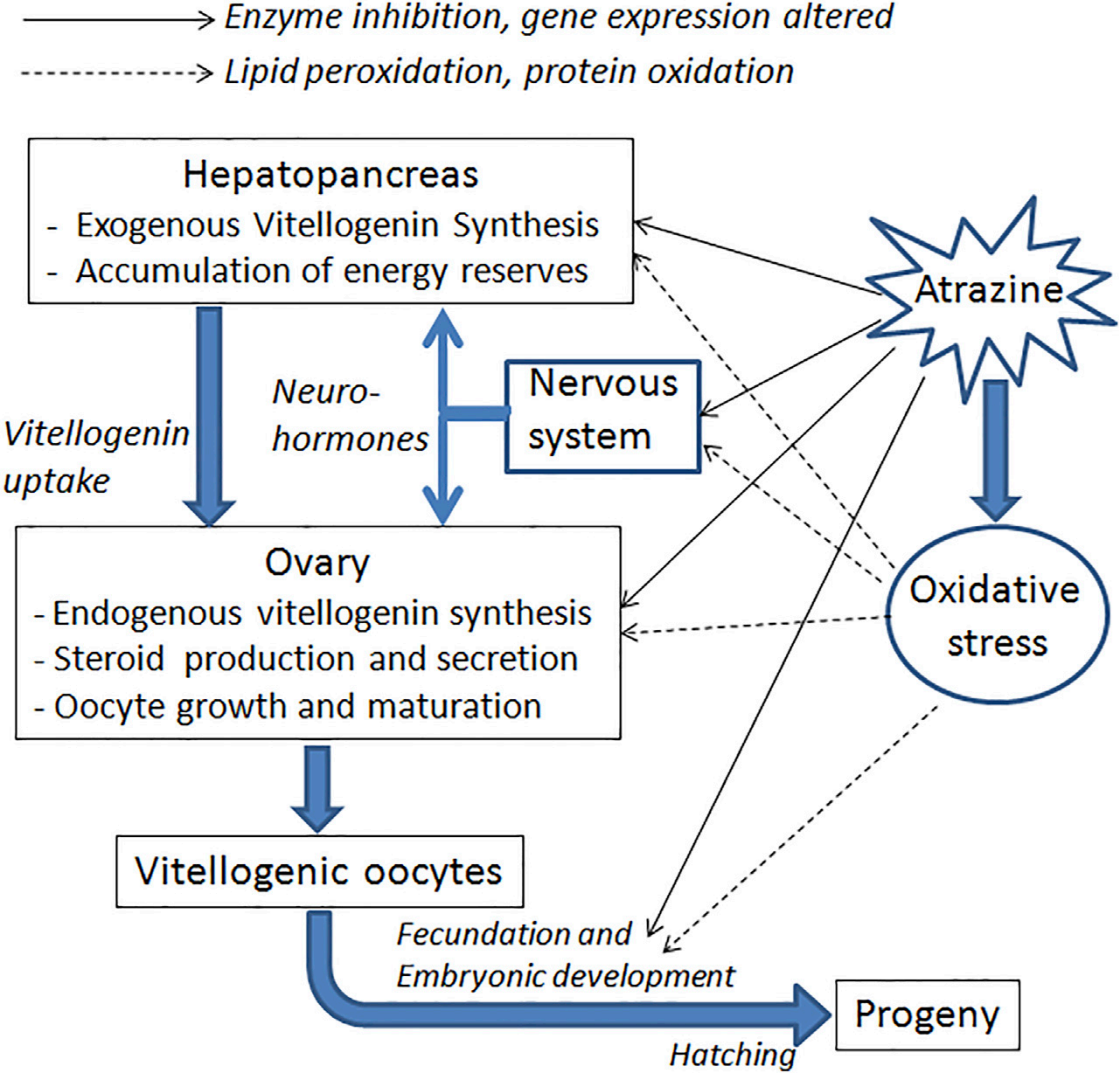
Schematic summary of the main targets and processes likely to be affected by atrazine, concerning crustacean reproduction. Both direct and indirect effects (by inducing oxidative stress) are indicated.

Inhibition of ovarian growth was also reported in the freshwater crayfish *Procambarus clarkii,* when exposed for 1 month to atrazine. This effect was observed in terms of both smaller oocytes and lower vitellogenin content in the ovary. Moreover, atrazine-exposed crayfish had a reduced expression of vitellogenin transcripts in both the ovary and the hepatopancreas (Silveyra et al., 2018). Down-regulation of several genes related to growth and the immune system has also been reported in tadpoles of *Xenopus laevis* (Langerveld et al., 2009). Figure 2 schematizes the possible pathways involved in these effects.

## Atrazine as an Endocrine Disruptor

An *in vivo* assay was conducted in intact, uni-eyestalk, and bi-eyestalk ablated females of *N. granulata* exposed for 30 days to 3 mg/L of atrazine (Silveyra et al., 2017). At the end of the assay, only the intact females showed a significant (*p* < 0.05) decrease in both the content of vitellogenic ovarian proteins and the proportion of vitellogenic oocytes, compared to the concurrent controls. Moreover, through *in vitro* assays made on the same species, a decrease in the proportion of vitellogenic oocytes was observed when atrazine was added to ovarian explants co-incubated with eyestalk tissue, but not when added to ovarian explants alone, suggesting that atrazine exerts an impairment on the secretion and/or action of one or more eyestalk neurohormones controlling ovarian growth (Silveyra et al., 2019).

On the other hand, an increase in circulating estradiol-like hormone was seen in *P. clarkii* exposed to atrazine, increasing the normal proportion of estradiol/testosterone (Figure 1B). This effect is potentially due to alterations in the activity and/or expression of the enzymes involved in gonadal steroidogenesis (Figure 2). In this respect, although some classical enzymes involved in the vertebrate steroidogenic pathway such as aromatase, have not been found in crustaceans, some others related to the cytochrome P450 (such as P450c17), as well as 5α-reductase have been found expressed in crustacean species (Thongbuakaew et al., 2016). In juveniles of the crayfish *Cherax quadricarinatus*, the sexual differentiation was affected by exposure to atrazine, increasing the relative proportion of females as the atrazine concentration increased (Mac Loughlin et al., 2016, Figure 1C). The latter effect suggests an alteration in the relative levels of sexual steroids (as observed in *P. clarkii*). However, some other possibilities are also plausible, in order to explain the observed effect of atrazine on sexual differentiation. In this respect, the androgenic gland of malacostracan crustaceans could be a possible target of atrazine. This endocrine gland has been reported as essential for the development and maintenance of both primary and secondary sexual characters of crustaceans (Barki et al., 2003, among others). Alternatively, atrazine could inhibit the hormonal secretion of the androgenic gland by affecting the secretion of the neurohormones that regulate the hormonal activity of this gland (Khalaila et al., 2002); some evidence of the interference of atrazine with neurohormone secretion has been obtained in the crab *N. granulata*, as mentioned above. Finally, a possible imbalance in the circulating levels of other hormones, such as methyl farnesoate, should not be discarded.

Methyl farnesoate, a juvenoid, has been characterized as a crustacean multifunctional hormone, involved in molting, reproduction, and metamorphosis, among other processes (Homola and Chang, 1997; Rodríguez et al., 2002). In daphnids, this hormone induces the production of males, under unfavorable environmental conditions (Olmstead and LeBlanc, 2003). The study of Dodson et al. (1999) in *Daphnia pulicaria* showed that exposure to atrazine during embryogenesis increases the proportion of males, at concentrations found in the environment. However, Palma et al. (2009a) reported a similar effect in *D. magna*, but only at higher atrazine concentrations, while at lower concentrations this herbicide showed to act as a juvenoid antagonist.

## Offspring Reduction and Teratogenic Effects

Regarding reproductive outcomes, exposure to successive generations of copepods to atrazine results in reduced offspring production (Bejarano and Chandler, 2003). In *D. magna*, several embryonic abnormalities such as the arrest of gastrulation, as well as underdeveloped spines and antennae, were noted after exposure to the herbicide, likely related to the antagonism of atrazine with 20-hydroxyecdysone, a hormone needed for successful embryonic development in daphnids (LeBlanc, 2007; Palma et al., 2009b). In *N. granulata*, larvae hatched from ovigerous females exposed to atrazine during the egg incubation period also showed several morphological abnormalities, such as hydropsy, hyperpigmentation, atrophy of spines and setae, and atrophy of eyes. (Figure 1D). Examples of teratogenic effects caused by atrazine on vertebrate species have been also reported; for instance, exposure of *X. laevis* tadpoles to atrazine during the organ morphogenesis produced several malformations in the intestine and skeletal muscle (Lenkowski et al., 2010).

In a more recent study, significant damage to DNA was observed in antennular cells of the crayfish *Faxonius virilis*, using the TUNEL assay (Abdulelah et., 2000). These data suggest that some of the teratogenic effects observed in crustaceans exposed to atrazine could be caused by damage to the genetic material of embryos (Figure 2).

## Oxidative Stress, Histopathological Effects, and Depletion of Energy Reserves

Finally, some effects of atrazine that could indirectly affect reproduction have also been reported. For instance, in the crayfish *Cherax destructor*, some hemolymphatic parameters, such as lactate and alkaline phosphatase levels, increased after after exposure to the herbicide, in correlation with histopathological effects on the hepatopancreas, and oxidative stress (Stara et al., 2018). These imbalances certainly have an impact on reproduction, since the hepatopancreas is a key organ for the synthesis of vitellogenin, which is ultimately taken up by the ovary (Figure 2). Some evidence of oxidative stress has also been reported in *P. clarkii* (Silveyra et al., 2018; Yang et al., 2021), as well as a decrease in the total hemocyte count (Yang et al., 2021), which represents inhibition of the immune system. On the other hand, a higher incidence of oocyte reabsorption has been reported in *N. granulata* females exposed during the ovarian re-maturation (Álvarez et al., 2015).

Atrazine exposure was also able to trigger oxidative stress responses and a decrease in lipid reserves in the shrimp *Palaemonetes argentinus*, therefore affecting the energy available for reproduction. A reduction in muscle glycogen content was observed in the crab *N. granulata* after chronic exposure to atrazine (Silveyra et al., 2017). In crayfish species, an increase in circulating lactate was also reported (Stara et al., 2018; Silveyra et al., 2018), indicating the metabolic effort triggered by the exposure to atrazine as a stressor. The oxidative stress response has been also reported in copepods, in terms of high production of reactive oxygen species, and the induction of conjugation enzymes; in addition, atrazine inhibited the expression of the ecdysteroid receptor, affecting the normal growth and molting of copepods (Yoon et al., 2019).

## Risk Mitigation

In a recent report, the Environmental Protection Agency (EPA, United States) has established a series of regulatory norms to reduce the risk potentially caused by atrazine, for both humans and wild species. Such regulations include the reduction of atrazine doses in residential areas, the improvement of personal protective equipment for atrazine handlers, several restrictions for aerial application along with drift reduction measures, as well as a series of label changes (EPA, 2020).

## Conclusion

In summary, multiple studies on a variety of crustaceans have provided a bulk of evidence about the negative effects of atrazine on the reproductive process at different levels. These include:

- Inhibition of ovarian growth.
- Endocrine disruption, through an imbalance in the status of the hormones controlling several reproductive aspects, including sexual differentiation.
- Offspring reduction, together with teratogenic effects and genotoxicity.
- Increased oxidative stress, reduction in energy reserves, and histopathological effects in tissues essential for reproductive success, such as hepatopancreas and ovary.
